# Supplementary material for: Space-time mapping relationships in sensorimotor communication during asymmetric joint action
Source: PeerJ. 2024 Jan 12;12:e16764. doi: 10.7717/peerj.16764 (PMC10789189; doi:10.7717/peerj.16764)
Supplement: Supplemental Information 1 [file peerj-12-16764-s001.docx]

**Space-Time Mapping Relationships in Sensorimotor Communication during Asymmetric Joint Action**

Ke Zhang^1^, Xin Tong^1^, Shaofeng Yang^1,2,3^, Ying Hu^1,2^, Qihan Zhang^1,2*^, Xuejun Bai^1,2*^

1 Department of Psychology, Tianjin Normal University, Tianjin, China

2 Academy of Psychology and Behavior, Tianjin Normal University, Tianjin, China

3 School of Psychology, Inner Mongolia Normal University, Hohhot, China

*Corresponding Author:

Xuejun Bai

Tianjin Normal University, Tianjin, 300387, China

Email address: [bxuejun@126.com](mailto:bxuejun@126.com)

Qihan Zhang

Tianjin Normal University, Tianjin, 300387, China

Email address: [zqhouou@126.com](mailto:zqhouou@126.com)

# SUPPLEMENTARY MATERIAL

# 1 Analysis results of keystroke response

## 1.1 Total motion time (TMT)

A 2 (task characteristic: distance, orientation) × 2 (cooperative intention: Coop, No-coop) × 4 (target: T1, T2, T3, T4) repeated measures ANOVA was conducted to analyze the total motion time from the pressing of starting key to the lifting of the target key, as presented in Figure S1. The results revealed a significant main effect of cooperative intention, *F*_(1,64)_=144.62, *p*<0.001, partial *η^2^*=0.69, as well as a significant main effect of target, *F*_(1.88, 120.13)_=56.98, *p*<0.001, partial *η^2^*=0.47. Furthermore, there was a significant interaction between cooperative intention and target, *F*_(1.84, 117.96)_=46.41, *p*<0.001, partial *η^2^*=0.42, and a significant interaction between task characteristic and target, *F*_(1.95, 124.94)_=23.27, *p*<0.001,partial *η^2^*=0.27. The triple interaction was also significant, *F*_(1.89, 121.05)_=13.49, *p*<0.001, partial *η^2^*=0.17. Subsequent simple effects analyses revealed the following patterns: Under the distance task without cooperative intention, the total motion time increased sequentially (*ps*< 0.001) for T1, T2, T3, T4. Under the distance task with cooperative intention, the total motion time of T1, T2, T3, and T4 increased sequentially (*ps* < 0.001). In the orientation task without cooperative intention, the total motion time of T1, T2, T4 was greater than T3 (*ps* < 0.05). In the orientation task with cooperative intention, the total motion time of T1 was less than T2, T3, T4 (*ps* < 0.05), indicating a trend of sequential increase for T1, T2, T3, and T4.


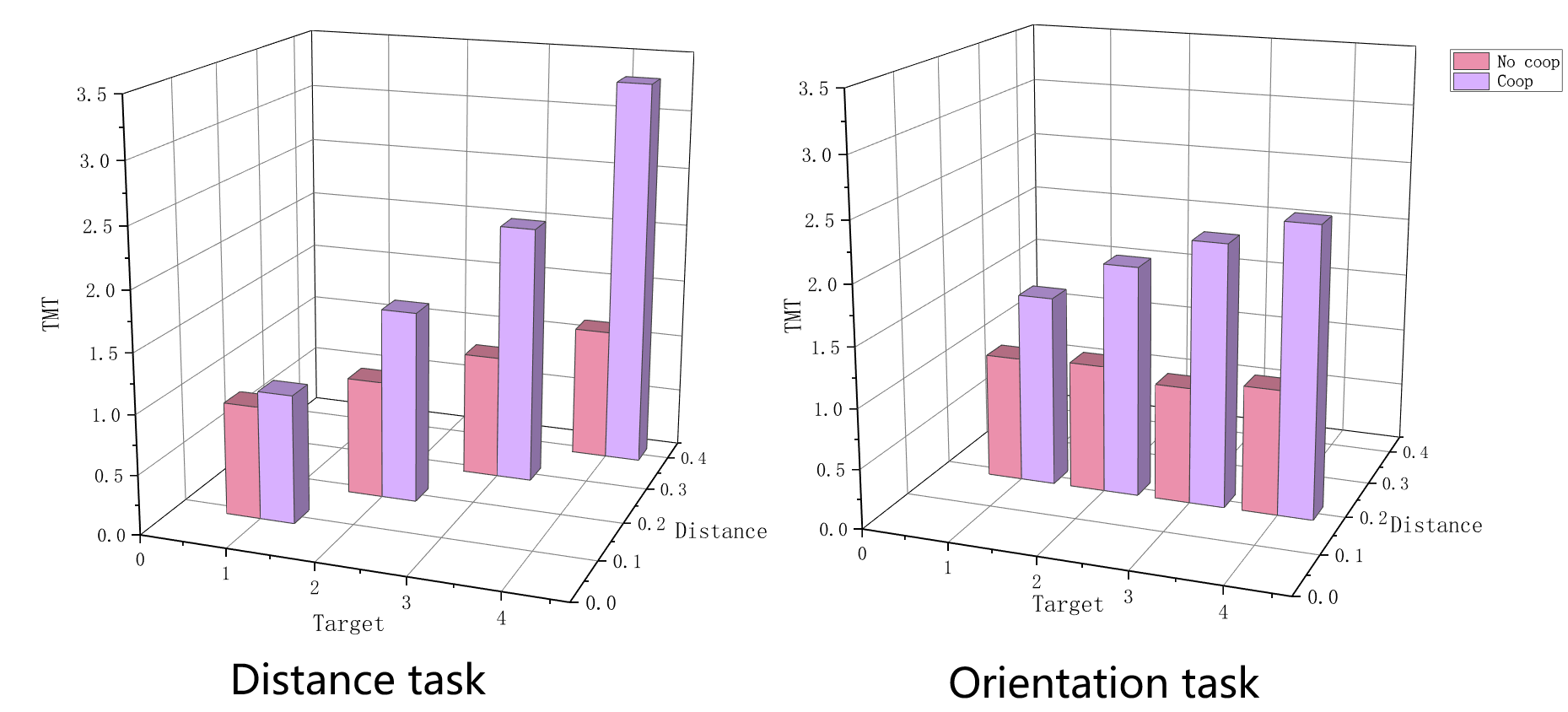


Figure S1 Total motion time in different experimental conditions

A 2 (task characteristic: distance, orientation) × 2 (cooperative intention: Coop, No-coop) repeated measures ANOVA was conducted on the signal-to-noise ratio (SNR_TMT_) to assess the quality of message communication related to TMT. The analysis revealed several significant findings: There was a significant main effect of cooperative intention, *F*_(1, 64)_ = 65.42, *p*<0.001, partial *η^2^* = 0. 51. A significant main effect of task characteristic was observed, *F*_(1, 64)_ = 52.10, p<0.001, partial *η^2^*= 0. 45. There was a significant interaction between cooperative intention and task characteristic, *F*_(1, 64)_ =11.18, *p*=0.001, partial *η^2^*= 0.15. Further simple effects analyses provided the following insights: SNR_TMT_ was significantly greater with cooperative intention than without cooperative intention for the distance task (*p*<0.001). SNR_TMT_ was significantly greater with cooperative intention than without cooperative intention for the orientation task (*p*= 0.001). In the Coop condition, SNR_TMT_ was significantly greater in the distance task than in the orientation task (*p*< 0.001). In the No-coop condition, SNR_TMT_ was significantly greater in the distance task than in the orientation task (*p*< 0.001), as illustrated in Figure S2.


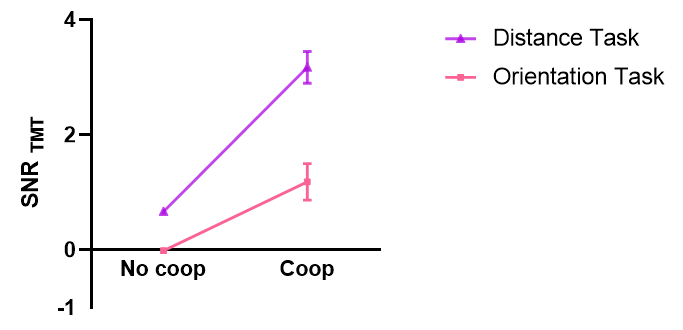


Figure S2 Quality of the message communication for total motion time SNR_TMT_ under different experimental conditions

## 1.2 Motion time (MT)

A 2 (task characteristic: distance, orientation) × 2 (cooperative intention: Coop, No-coop) × 4 (target: T1, T2, T3, T4) repeated-measures ANOVA was conducted on the motion time from the starting key lift to the target key press, as depicted in Figure S3. The results yielded several significant findings: There was a significant main effect of cooperative intention, *F*_(1,64)_=16.19, *p*<0.001, partial *η^2^*=0.20. A significant main effect of task characteristic was observed, *F*_(1.64)_ =4.84, *p*<0.05, partial *η^2^*=0.07. A significant main effect of target was found, *F*_(2.42,154.76)_ = 36.24, *p*<0.001, partial *η^2^*=0.36. An interaction between cooperative intention and target was present, *F*_(2.13, 136.17)_ = 3.88, *p*<0.05, partial *η^2^*=0.06. Further simple effects analyses provided the following insights: Without cooperative intention, MT of T1 was smaller than T2, T3, and T4, and MT of T2 and T3 was smaller than T4 (*ps* < 0.05). With cooperative intention, MT of T1 was smaller than T2, T3, and T4 (*ps* < 0.05). Additionally, the interaction between task characteristic and target was significant, *F*_(1.91, 122.17)_=37.02, *p*<0.001, partial *η^2^*=0.37. Simple effects analysis revealed that under the distance task, the MT of T1, T2, T3, and T4 increased sequentially (*ps* < 0.05), and under the orientation task, the MT of T1 was greater than T3 (*p* < 0.05).


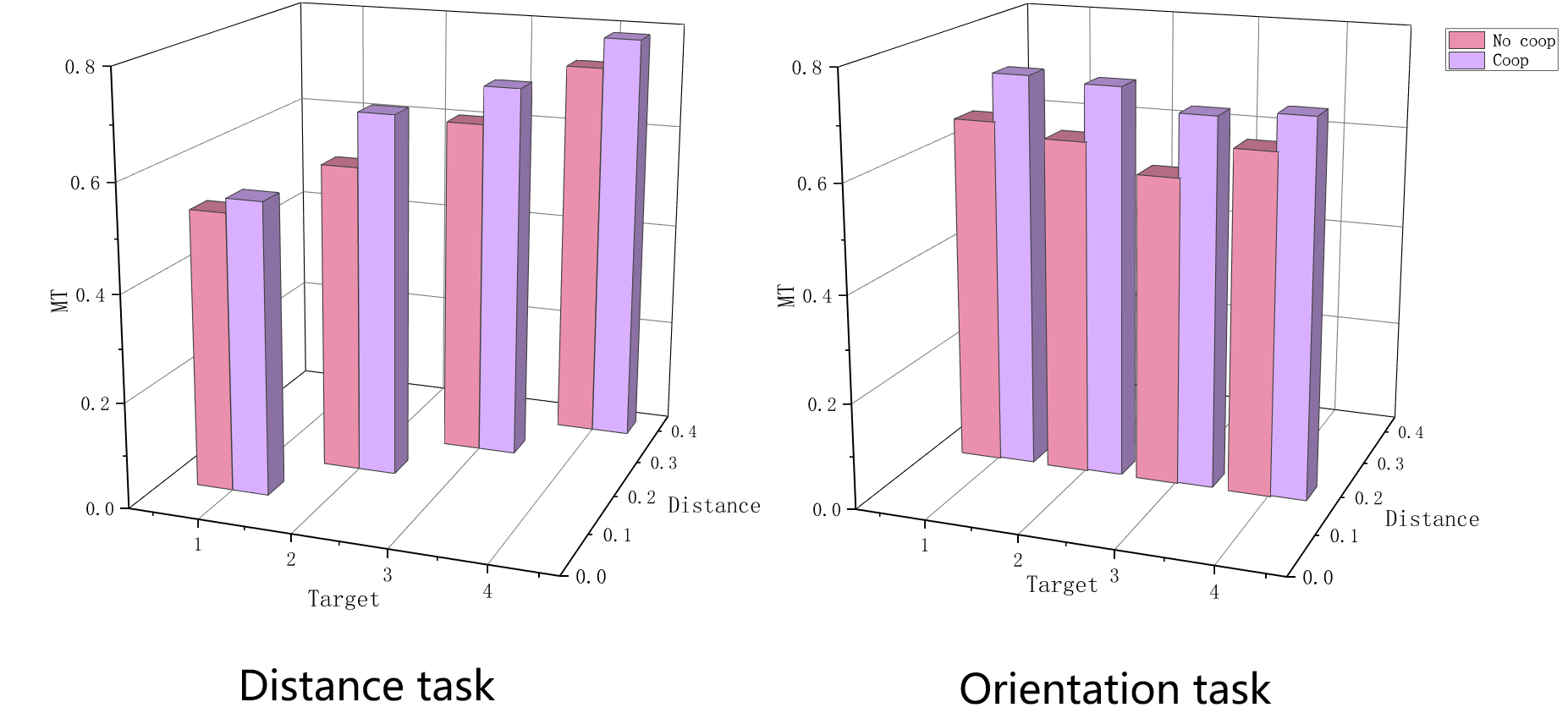


Figure S3 Motion time in different experimental conditions

A 2 (task characteristic: distance, orientation) × 2 (cooperative intention: Coop, No-coop) repeated measures ANOVA was conducted on the quality of message communication during the motion time (SNR_MT_), as depicted in Figure S4. The analysis revealed a significant main effect of task characteristic, *F*_(1, 64)_ = 210.71, *p*<0.001, partial *η^2^* = 0.77. Simple effects analysis indicated that SNR_MT_ was significantly greater for the distance task than for the orientation task (*p*< 0.001).


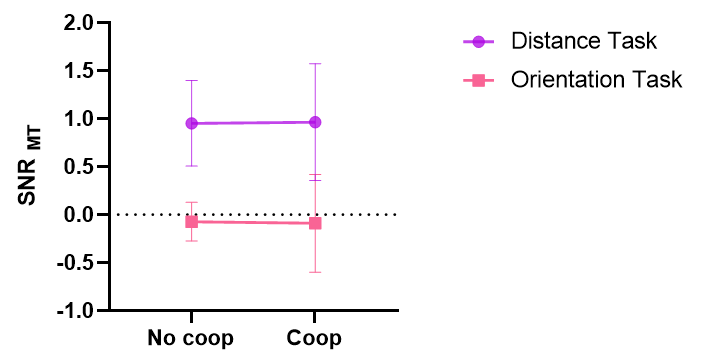


Figure S4 Quality of the message communication of motion time SNR_MT_ for different experimental conditions

# 2 Statistical results based on Bayesian repeated measures ANOVA

## 2.1 Keystroke response

### 2.1.1 Total motion time (TMT)

A Bayesian repeated-measures ANOVA of 2 (task characteristic: distance, orientation) × 2 (cooperative intention: Coop, No-coop) × 4 (target: T1, T2, T3, T4) was conducted on the total motion time from the starting key press to the target key lift. The analysis revealed extremely strong evidence supporting main effects of cooperative intention (BF_incl_= ∞), target (BF_incl_=∞), and task characteristic (BF_incl_= ∞). Additionally, there was strong evidence for an interaction between cooperative intention and task characteristic (BF_incl_ =7.50×10^7^), an interaction of cooperative intention and target (BF_incl_=∞), and an interaction of task characteristic and target (BF_incl_= ∞) and the presence of triple interaction (BF_incl_ =5.51×10^8^). Subsequent Bayesian paired-sample t-tests were performed. The results indicated the following: In the distance task without cooperative intention, there was strong evidence that the total motion time (TMT) for T1, T2, T3, and T4 increased sequentially. TMT for T1 was smaller than T2 (BF_10_ = 1.675×10^8^), T3 (BF_10_ = 3.176 × 10^15^), T4 (BF_10_ = 8.293 × 10^12^). TMT for T2 was smaller than T3 (BF_10_ = 8.616 × 10^11^), T4 (BF_10_ = 8.894 × 10^9^), and TMT for T3 was smaller than T4 (BF_10_ = 35648.367). In the distance task with cooperative intention, there was strong evidence that TMT for T1, T2, T3, and T4 increased sequentially. T1's TMT was smaller than T2 (BF_10_=4.654×10^8^),T3 (BF_10_=3.061×10^11^),T4(BF_10_=3.804×10^11^). T2's TMT is smaller than T3 (BF_10_=9.378×10^7^), T4(BF_10_=3.063×10^10^), and T3's TMT is smaller than T4 (BF_10_=4.222×10^6^). In the orientation task without cooperative intention, there was evidence that T3's TMT was smaller than T1 (BF_10_=832.513), T2 (BF_10_=40.455), T4 (BF_10_=713.907). In the orientation task with cooperative intention, there was a tendency for TMT of T1, T2, T3, and T4 to increase sequentially, with T1's TMT being smaller than T2 (BF_10_=11.127), T3 (BF_10_=13.062), T4 (BF_10_=65.054), and T2's TMT is smaller than T4 (BF_10_=3.496).

A 2 (task characteristic: distance, orientation) × 2 (cooperative intention: Coop, No-coop) Bayesian repeated-measures ANOVA was conducted on the quality of message communication for total motion time (SNR_TMT_). The analysis revealed extremely strong evidence supporting the existence of a main effect of cooperative intention (BF_incl_ = 2.43×10^10^), a main effect of task characteristic (BF_incl_ = 9.45 × 10^7^), and an interaction between cooperative intention and task characteristic (BF_incl_=446.69). Subsequent Bayesian paired-sample t-tests were performed between the different conditions, yielding the following results: SNR_TMT_ was greater in the distance task with cooperative intention than without cooperative intention (BF_10_=1.255×10^10^). In the orientation task, SNR_TMT_ was greater with cooperative intention than without cooperative intention (BF_10_=59.607). SNR_TMT_ was greater in the distance task than in the orientation task without cooperative intention (BF_10_=5.864×10^15^). SNR_TMT_ was greater in the distance task than in the orientation task with cooperative intention (BF_10_=9724.997).

### 2.1.2 Dwell time (DT)

A Bayesian repeated-measures ANOVA of 2 (task characteristic: distance, orientation) × 2 (cooperative intention: Coop, No-coop) × 4 (target: T1, T2, T3, T4) was conducted on the dwell time of the target key under different experimental conditions. The analysis provided strong evidence supporting various main effects and interactions, as follows: a main effect of cooperative intention (BF_incl_=7.15×10^13^), a main effect of target (BF_incl_ =7.15×10^13^), a main effect of task characteristic (BF_incl_ =6.17 ×10^7^), an interaction of cooperative intention and task characteristic (BF_incl_ =1.49×10^6^), and an interaction of cooperative intention and target (BF_incl_ =4.34× 10^14^), and an interaction of task characteristic and target (BF_incl_ =3.17×10^8^), and a triple interaction (BF_incl_=1.04×10^7^). Subsequent Bayesian paired-sample t-tests were performed to compare different conditions. The results were as follows: In the distance task without cooperative intention, the DT for T1 (BF_10_ = 66105.992), T2 (BF_10_ = 1.56 × 10^6^), and T3 (BF_10_ = 2.58 × 10^9^) were all greater than T4. In the orientation task without cooperative intention, T1's DT was smaller than T2 (BF_10_=34616.354), T3 (BF_10_=900.615), T4 (BF_10_=35676.102). In the distance task with cooperative intention, the DT of T1, T2, T3, T4 was increasing in the order. T1's DT was smaller than T2 (BF_10_= 657715.261), T3 (BF_10_ = 8.383 × 10^9^), T4 (BF_10_ = 1.852 × 10^10^). T2's DT was smaller than T3 (BF_10_ = 37742.363), T4 (BF_10_=7.337 ×10^8^), and T3's DT was smaller than T4 (BF_10_=1.591×10^6^). In the orientation task with cooperative intention, the dwell time for T1, T2, T3, and T4 showed a tendency to increase sequentially. The DT of T1 was smaller than T2 (BF_10_=10.043), T3 (BF_10_=235.369), T4 (BF_10_=1365.348), and the DT of T2 was smaller than T4 (BF_10_=86.502). Additional comparisons included: The dwell time of T1 in the distance task without cooperative intention was greater than in the orientation task without cooperative intention (BF_10_=656.175). The dwell time of T4 in the distance task without cooperative intention was less than in the orientation task without cooperative intention (BF_10_=7.614). The dwell time of T1 in the distance task with cooperative intention was less than in the orientation task with cooperative intention (BF_10_=5.274). The dwell time of T4 in the distance task with cooperative intention was greater than in the orientation task with cooperative intention (BF_10_=49.734).

A 2 (task characteristic: distance, orientation) × 2 (cooperative intention: Coop, No-coop) × 4 (target: T1, T2, T3, T4) Bayesian repeated-measures ANOVA was conducted on the variability of dwell time for the target key (SD_DT_). The analysis provided extremely strong evidence supporting various effects, as follows: a main effect of cooperative intention (BF_incl_=∞), a main effect of target (BF_incl_=∞), and an interaction of cooperative intention and target (BF_incl_ =∞).

A 2 (task characteristic: distance, orientation) × 2 (cooperative intention: Coop, No-coop) Bayesian repeated measures ANOVA conducted on the quality of message communication measured by target key dwell time SNR_DT_, the results indicated the following: There was extremely strong evidence supporting a main effect of cooperative intention (BF_incl_=∞), a main effect of task characteristic(BF_incl_=441831.08), and an interaction of cooperative intention and task characteristic (BF_incl_ = 278041.78). Furthermore, Bayesian paired-sample t-tests revealed that SNR_DT_ was higher in the distance task with cooperative intention compared to without (BF_10_=7.133×10^13^). In the orientation task, SNR_DT_ was higher with cooperative intention than without (BF_10_=107.997). Additionally, SNR_DT_ for the distance task without cooperative intention was lower than for the orientation task without cooperative intention(BF_10_=1.877×10^7^), and SNR_DT_ for the distance task with cooperative intention was higher than for the orientation task with cooperative intention (BF_10_=234.273).

### 2.1.3 Motion time (motion time, MT)

A 2 (task characteristic: distance, orientation) × 2 (cooperative intention: Coop, No-coop) × 4 (target: T1, T2, T3, T4) Bayesian repeated-measures ANOVA was conducted on the motion time. The analysis revealed the following results: There was extremely strong evidence supporting a main effect of cooperative intention (BF_incl_ = 114.42), a main effect of target (BF_incl_ = 8.04 × 10^13^), a main effect of task characteristic (BF_incl_=8.04×10^13^), and an interaction of task characteristic and target (BF_incl_ = 4.76 ×10^14^).

A 2 (task characteristic: distance, orientation) × 2 (cooperative intention: Coop, No-coop) Bayesian repeated-measures ANOVA was performed on the quality of the message communication for motion time SNR_MT_. There was extremely strong evidence in the current data to support a main effect of task characteristic (BF_incl_=1.20×10^15^).

## 2.2 Motion trajectories

A 2 (task characteristic: distance, orientation) × 2 (cooperative intention: Coop, No-coop) × 4 (target: T1, T2, T3, T4) Bayesian repeated-measures ANOVA was conducted on the maximum movement height MAX_MH_. The analysis yielded the following results: There was very strong evidence supporting a main effect of cooperative intention (BF_incl_=175.00), a main effect of targets (BF_incl_=∞), a main effect of task characteristic (BF_incl_=∞), an interaction of cooperative intention and target (BF_incl_=18.21), and an interaction of task characteristic and target (BF_incl_=∞). There was weaker evidence supporting the presence of the interaction of cooperative intention and task characteristic (BF_incl_ = 1.61). There was moderate evidence supporting the presence of the triple interaction (BF_incl_= 8.57). Subsequent Bayesian paired-sample t-tests revealed the following significant comparisons: For the distance task without cooperative intention: MAX_MH_ for T1, T2, T3, T4 increased in order. MAX_MH_ of T1 was less than T2 (BF_10_ = 1.957 × 10^29^), T3 (BF_10_ = 3.127 × 10^31^), T4 (BF_10_ = 8.621 × 10^29^). MAX_MH_ of T2 was smaller than T3 (BF_10_=9.341×10^26^), T4 (BF_10_=1.349×10^25^), and MAX_MH_ of T3 was smaller than T4 (BF_10_=3.185×10^14^). For the distance task with cooperative intention: The MAX_MH_ of T1, T2, T3, T4 were increase in order. The MAX_MH_ of T1 was smaller than T2 (BF_10_=1.067× 10^13^), T3 (BF_10_=4.387×10^23^), T4 (BF_10_=9.440×10^23^). T2's MAX_MH_ was less than T3 (BF_10_=1.683×10^18^), T4 (BF_10_=5.603×10^14^), and T3's MAX_MH_ was less than T4 (BF_10_=109.232). MAX_MH_ of T1 (BF_10_=109.232), T2 (BF_10_=109.232), and T3 (BF_10_=109.232) in the distance task with cooperative intention was greater than that without cooperative intention. For the orientation task without cooperative intention: The MAX_MH_ of T3 was smaller than T1 (BF_10_=594199.662), T2 (BF_10_=68.577), T4 (BF_10_=1.067×10^13^), and the MAX_MH_ of T2 was smaller than T4 (BF_10_=4.007). For the orientation task with cooperative intention: The MAX_MH_ of T3 was smaller than T1 (BF_10_=24.125), T2 (BF_10_=56.466), T4 (BF_10_=2.286×10^7^), and the MAX_MH_ of T1 was smaller than T4 (BF_10_=322.528). The MAX_MH_ of T2 (BF_10_=15.094), T4 (BF_10_=9.909) under the orientation task with cooperative intention was larger than that of the without cooperative intention.
